# Supplementary material for: Are carbon emissions trading and green financial instruments synergistic? -Comprehensive quantitative research based on content analysis
Source: PLoS One. 2024 Mar 7;19(3):e0298601. doi: 10.1371/journal.pone.0298601 (PMC10919601; doi:10.1371/journal.pone.0298601)
Supplement: S1 Data — (ZIP) [file pone.0298601.s001.zip › Data/地级市Z_F工作报告.zip!/╘╞─╧/╟·╛╕╩╨/└┤╫╘╣1⁄2╓┌║┼í╛┬φ┐╦╩2╛▌═°í┐_2017.docx]

各位代表：

　　我代表市人民政府，向大会报告工作，请予审议，并请市政协委员和列席人员提出意见。

　　一、2016年工作回顾

　　2016年，是全市上下在困难复杂的经济环境中攻坚克难、各项工作取得显著成效的一年。在市委的坚强领导下，市人民政府认真贯彻落实党中央、国务院和省委、省政府的决策部署，出台了一批稳增长的政策措施，谋划了一批利长远的重大举措，全面完成了市四届人大五次会议确定的目标任务，圆满实现了“十三五”开门红。预计实现市内生产总值1765亿元，增长9%以上；规模以上固定资产投资1792亿元，增长30%；一般公共预算收入126.3亿元，增长7%；社会消费品零售总额565亿元，增长12.5%；城镇和农村常住居民人均可支配收入分别为29539元、10490元，分别增长9%、11%；单位生产总值能耗下降3%；城镇登记失业率为3.5%；居民消费价格上涨1.6%。

　　一年来，我们狠抓政策落地，促进了经济运行稳中向好。认真贯彻落实中央、省关于稳增长和供给侧结构性改革的工作部署，出台稳增长开好局38条政策和工业企业帮扶10条措施，实行经济运行年计划、季分析、月调度工作机制，稳定一产、帮扶二产、做强三产。拨付各级财政奖补资金15.6亿元，关闭退出煤矿矿井42对、化解煤炭过剩产能757万吨，淘汰钢铁落后产能123万吨。出台促进房地产业发展的13条措施，建立个体工商户和农民工住房公积金缴纳制度，发放住房公积金贷款28.7亿元，棚户区改造货币化安置比例达83.1%，化解商品房库存371万平方米。降低企业用电、物流成本21.3亿元，减轻企业税费负担15.1亿元，促成180户企业与银行签订贷款协议628.9亿元，帮助130余户企业实现债务重组84.1亿元，新增小微企业1.04万户，新纳规升规企业81户，推动120户停产减产企业复产达产，非公经济增加值增长10%以上，规模以上工业增加值增长7.5%以上。一般公共预算支出突破400亿元，保障了一批补短板项目的顺利实施。筹集项目前期工作经费7.8亿元，争取国家专项建设基金和省重点项目投资基金80.6亿元、国家和省项目补助资金25.8亿元，集中开工重点项目198个，一批带动力强的重大项目落地生根、开花结果。

　　一年来，我们聚焦重点产业，推动了产业转型升级初见成效。结合实际确定了重点发展的6大产业，出台实施意见和五年行动计划，积极构建现代产业体系。着力增强优质农产品供给能力。实现粮食产量340.6万吨。建成猕猴桃、玫瑰等4个万亩特色产业示范基地。会泽阿穆尔鲟鱼鱼子酱投产，威毅垅田马铃薯深加工、联想佳沃蓝莓分选加工和冷链物流中心项目开工建设。广东温氏、德康希望等养殖项目进展顺利，新创建“百千万”畜禽养殖示范场37个。新增省级农业龙头企业12个、市级以上农业合作示范社51个。加快工业转型升级步伐。今飞汽车轮毂一期项目实现当年建设、当年投产。液态金属电子手写笔和油墨生产线等项目建成投产。昆明冶研新材料电子级多晶硅实现稳定生产。众一化工永固紫实现批量生产，米高集团农用硝酸钾、云隆化工等项目加快推进。一汽红塔蓝舰皮卡汽车成功发布。坚定不移推动煤炭产业转型升级，复产复建矿井136对，生产原煤2500万吨。优化园区空间布局，完成麒麟、沾益、马龙工业园区更名并启动规划修编工作，沾益、师宗工业园区进入省级高新区行列，曲靖经开区被确定为长江经济带国家级转型升级示范区。推动现代服务业提速升级。万达城市综合体、陆良农村商业模式创新示范园等20个商贸物流项目加快推进。建成电子商务县级运营服务中心8个、乡镇和村级服务站615个。富源营上镇全省“七彩乡村购物”试点初见成效。在全省率先组建旅游产业发展基金，设立旅游发展研究院。启动高速公路旅游标识标牌安装工作，沾益珠江源、罗平金鸡峰丛、麒麟水乡等重点旅游项目加快推进。接待国内外旅游者1729万人次，实现旅游业总收入143亿元。成功举办国际女足赛、全国男子篮球联赛等国际国内赛事活动100余场次，有力提升了城市形象。全力构建科技创新体系。建成国家级科技孵化器1个、众创空间3个、省级重点实验室1个，认定国家高新技术企业32户。湖南农业大学油菜全产业链科技项目稳步推进。成功举办中国第二届液态金属产业技术高峰论坛。清华启迪众创空间和科技园项目落户曲靖。新增国家驰名商标1件、云南名牌产品6个。

　　一年来，我们着力补短板，实施了一批打基础利长远的重大工程。完成公路基础设施投资200.1亿元。曲陆高速改扩建工程建成通车，东过境、宣曲、沾会、大瓦仓至昌隆铺、江召新建段高速公路建设加快推进，沾益至寻甸、富源至兴义、召夸至泸西高速公路启动建设，三宝至清水、宣威至威宁、罗平至八大河高速公路前期工作稳步推进。新建、改建农村公路2495公里。完成铁路建设投资21.4亿元。全市人民翘首以盼的沪昆高铁建成通车，曲靖北站、富源北站及曲靖主城区K1公交快线同步投入使用。渝昆高铁、师宗至文山铁路前期工作进展顺利。完成航空网建设投资7000万元。宣威支线机场选址报告通过国家民航局评审。沾益、会泽、罗平、马龙通用机场前期工作有序开展，罗平、麒麟水上飞机实现验证飞行。完成水利投资65亿元。阿岗水库开工建设，车马碧水库可研报告获批，黑滩河水库纳入国家“十三五”规划。续建及新开工骨干水源工程35件，全面完成586件小型病险水库除险加固工程，新建农田水利工程3.2万件，改造中低产田地51.8万亩。完成能源投资200亿元。建成风电场7座、输变电工程11项、输电线路171.8公里、城市天然气管网84.7公里，中心城区新增天然气用户1.9万户。完成信息网建设投资23亿元。实现光纤宽带100%接入行政村、4G网络100%覆盖行政村目标。完成曲靖市新型智慧城市总体规划编制，平安曲靖数据化系统、电子政务系统等一批信息化应用项目投入使用。

　　一年来，我们统筹城乡，加强了城乡规划建设管理。完成城市总体规划、土地利用总体规划和轨道交通、综合管廊、海绵城市等专项规划编制。谋划推进麒沾马一体化工程和城市公园项目38个，改造提升中心城区道路21.7公里，打通断头路13.1公里。全面放开城镇落户限制，沾益成功撤县设区，师宗、马龙县城基础设施投融资体制改革试点有序推进，新型城镇化试点通过国家中期评估，全市户籍人口城镇化率达35.7%。启动建设地下综合管廊25.4公里，开工建设农村危房改造及抗震安居工程10.05万户、棚户区改造1.16万户，完成公租房“以购代建”2248套。开展城市“四治三改一拆一增”和农村“七改三清”环境整治行动，拆除违法违规建筑41.3万平方米，完成乡镇乡村清洁工程20个。生态文明建设成效明显。中央环保督察组反馈问题逐项分解整改，群众投诉举报问题全部办结。建成城乡生活污水处理设施和垃圾填埋场83个、城市污水管网81.8公里，中心城区环境空气质量优良率达97.8%。完成营造林69.3万亩，森林覆盖率达46%。

　　一年来，我们服务民生，办成了一批事关群众切身利益的实事。出台坚决打赢脱贫攻坚战的决定，扎实开展“挂包帮”“转走访”活动，三千单位包乡村、八万干部下基层，675支驻村扶贫工作队投身脱贫攻坚主战场。组建总规模达143亿元的曲靖浦发扶贫投资发展基金。投入各类扶贫资金181.4亿元，其中，各级财政专项扶贫资金19.1亿元、整合部门资金101.6亿元、金融扶贫资金55.2亿元、各类帮扶资金5.5亿元。大力实施“五个一批”脱贫工程，圆满完成全市10个贫困乡、133个贫困村出列，14.88万贫困人口脱贫的任务，罗平县脱贫摘帽完成初审，会泽县、宣威市脱贫攻坚取得突破，实现脱贫攻坚首战告捷。启动区域教育中心建设，完成“全面改薄”学校200所，新建市、县中心城区中小学12所、标准化幼儿园12所，曲靖一中卓立学校建成投入使用，市民中迁建、北大附中曲靖经开区实验学校竣工。麒麟、沾益、马龙县域义务教育基本均衡通过国家督导评估验收。高考再创佳绩。启动区域医疗卫生中心建设，市妇幼保健院新院、市中医院新院等项目有序推进，新建、改扩建乡镇卫生院4个、村卫生室156个。成功创建三级甲等医院5家。实现省内异地就医联网结算。开展青少年儿童“先心病”筛查2.2万人、治愈152人，救助白血病患儿31例。启动公立医院改革试点工作，调整医疗服务价格，取消市级医院药品加成，基本药物制度不断完善，基层中医药服务能力提升工程通过评审验收。“全面两孩”政策得到落实。启动国家公共文化服务体系示范区创建，市美术馆、图书馆、博物馆、文体中心实现免费对外开放。曲靖籍田径运动员蔡泽林荣获里约奥运会银牌、游泳运动员邹连康荣获里约残奥会3金1银。就业形势保持稳定，发放创业担保贷款16.4亿元，扶持创业1.6万人（户），新增城镇就业3.9万人，农村劳动力转移就业和劳务输出14.6万人。各类社会保险参保人数达525.9万人次，新农合参合率达98.1%，大病保险补偿8710.1万元。发放城乡低保、临时救助等资金12.5亿元。新建、改扩建敬老院16所，建成居家养老服务中心48个。建成省级民族团结进步示范村（社区）8个、民族特色村3个。会泽地震灾区灾后恢复重建工作扎实推进。殡葬改革稳妥推进。

　　一年来，我们深化改革开放，激发了各类市场主体竞相迸发的活力。取消和调整行政许可项目15项，清理行政审批中介服务事项85项，制定并公布市、县、乡三级权责清单，市、县两级投资审批中介超市联网运行，投资项目在线审批监管平台投入使用。全力推进商事制度改革，颁发全省首张“五证合一、一照一码”营业执照，新增市场主体4.6万户。在全省率先建成企业信用信息管理系统，清理公布随机抽查事项348项，“双随机、一公开”工作扎实推进。“营改增”试点全面推开，国税、地税征管体制改革工作成效明显。新增银行业金融机构5家、“新四板”挂牌企业2户，实现银行间市场及债券市场融资22亿元。全国农田水利改革现场会在曲靖召开。新增土地承包经营权流转32万亩，流转林地22.4万亩。全面完成城镇周边永久基本农田划定，不动产统一登记体系初步建立。努力扩大对内对外开放，与深圳缔结为友好城市，与金华等友城交流合作进一步加强。昆明至深圳高铁“曲靖号”文化旅游专列正式开通。积极参加第四届南博会等交流活动，突出珠三角、长三角、环渤海等重点地区开展招商活动，到位市外国内资金912亿元，增长10.6%。外贸进出口总额完成6.3亿美元，增长12%。

　　一年来，我们强化治理，保持了社会安定和谐的良好局面。严格落实安全生产责任制，深入开展重点行业、重点领域安全生产专项整治和打非治违专项行动，各类事故起数、死亡人数、受伤人数、直接经济损失分别下降23.1%、3.9%、20.9%、17.5%，实现安全生产“一杜绝、四下降”目标。启动国家食品安全城市创建工作，地方政府对食品安全负总责试点顺利推进。启动第四轮禁毒防艾人民战争，立体化社会治安防控体系初步建成，保持对刑事犯罪高压态势，人民群众安全感不断增强。“七五”普法顺利启动，社区矫正和安置帮教工作得到加强。严格落实属地管理责任，一批矛盾纠纷和信访积案得到有效化解，到京非访和到省集体上访批次和人数大幅下降。

　　一年来，我们开拓进取，树立了敢于担当狠抓落实的良好形象。扎实开展“两学一做”学习教育。认真执行市人大及其常委会的决议决定，自觉接受市人大的法律监督、工作监督，主动接受市政协的民主监督，157件人大代表议案、建议和320件政协提案全部办结，满意率达100%。严格执行重大行政决策程序规定，政府法律顾问制度普遍建立，法治政府建设取得实效。“三公经费”下降7.8%。完成公共资源交易3958项，交易总额147亿元。党政机关公务用车制度改革走在全省前列。推进审计监督全覆盖，查出违规金额5.3亿元，促进增收节支4.2亿元，核减工程投资13.9亿元。扎实开展“狠抓落实年”活动，整治“不作为、乱作为”突出问题525个，问责干部324人，政纪处分199人。

全力支持各民主党派、工商联、无党派人士参政议政，全力支持工会、共青团、妇联等人民团体发挥作用。国防动员和后备力量建设不断加强，“曲靖舰”入列南海舰队，双拥共建工作取得新成果。国家安全、广播电视、侨务、档案、保密、史志、应急、地震、移民、水文、邮政、统计和统计调查等工作取得新成绩，妇女、儿童、青少年、慈善等事业取得新进步。

　　各位代表，过去的一年，成绩来之不易，这是省委、省政府和市委坚强领导、科学决策的结果，是市人大、市政协有力监督、倾力支持的结果，是全市广大干部群众团结奋斗、狠抓落实的结果。在此，我代表市人民政府，向全市广大干部群众，向人大代表、政协委员、各民主党派、工商联和无党派人士、各人民团体、离退休老干部、社会各界人士，向驻曲部队指战员、武警官兵和中央、省属单位，向所有关心支持曲靖改革发展的同志们，致以崇高的敬意，并表示衷心的感谢！

　　成绩已成过去，未来更具挑战。我们也清醒地看到前进中的问题和不足，主要表现在：传统产业转型升级缓慢，新兴产业发展不足，工业和民间投资增速下滑，实体经济困难较多，经济稳定增长的基础还不牢固；改革开放相对滞后，科技创新能力不足，人才支撑作用亟待提高，经济增长内生动力不足；城乡基础设施薄弱，少数重点项目推进缓慢，改善生态环境质量的任务仍然较重；社会事业欠账较多，脱贫攻坚任务艰巨；生产安全、食品安全、社会安全领域基础薄弱，部分干部群众法治意识不强，维护社会安全稳定任重道远；各级政府和部门破解发展难题的能力亟待增强，部分干部主动干事创业的活力还未有效激发，依法行政的素质和能力尚需提高。对此，我们将在今后工作中采取更有针对性、更有力的措施，切实加以解决。

　　二、2017年工作安排

　　2017年，我们将迎来党的十九大隆重召开，同时也是全面贯彻落实省第十次党代会精神的开局之年、供给侧结构性改革的深化之年、推动“十三五”转型发展的关键之年。我们既要看到，国际国内经济形势复杂多变，不确定因素增多，仍经受着经济下行压力加大、竞争优势不足的严峻考验。我们更要看到，国家出台了一系列重大政策措施，省第十次党代会对全省跨越式发展提出了更高要求，曲靖的产业基础、区位交通、资源环境等潜力正在释放，我们具备加快发展的基础和条件。只要我们始终保持开拓创新的锐气、敢为人先的勇气、持续攻坚的士气，就一定能推动经济社会发展再上新台阶、实现新跨越。

　　政府工作的总体要求是：全面贯彻党的十八大和十八届三中、四中、五中、六中全会精神，深入贯彻习近平总书记系列重要讲话和考察云南重要讲话精神，认真落实中央经济工作会和农村工作会、省第十次党代会、市第五次党代会精神，以及省委陈豪书记赴曲靖调研重要讲话精神，统筹推进“五位一体”总体布局和“四个全面”战略布局，牢固树立和落实新发展理念，紧紧围绕“三大定位”，坚持稳中求进、好中求快，以提高发展质量和效益为中心，以深化供给侧结构性改革为主线，紧盯“生产总值突破2000亿元”奋斗目标，始终坚持稳增长、促改革、调结构、惠民生、优环境、防风险，深化“狠抓落实年”活动，在传统产业转型、新兴产业培育、创新驱动、“三大战役”、脱贫攻坚、教育卫生区域中心建设、生态文明建设、社会治理、全面从严治党等方面取得重大突破，全力推动经济社会跨越发展。

　　经济社会发展的主要预期目标建议为：市内生产总值增长9%以上，规模以上固定资产投资增长25%以上，一般公共预算收入增长7%以上，社会消费品零售总额增长12%以上，城镇和农村常住居民人均可支配收入分别增长8%以上、10%以上，居民消费价格涨幅控制在3%左右，城镇登记失业率控制在4%以内，单位生产总值能耗完成省下达的目标任务。

　　重点抓好8项工作：

　　（一）加快产业转型升级，保持经济平稳较快增长

　　全力稳住经济运行基本面。坚持稳中求进的工作总基调，以五大发展理念为统领，进一步完善政策措施，稳定发展一产、优化提升二产、加快发展三产，努力培育新的经济结构。坚持把工业经济作为稳增长的主战场，打好工业经济攻坚战，确保规模以上工业增加值增长8%以上。强化对重点行业、重点企业的精准服务，推动停产减产企业尽快恢复生产、复产企业尽快达产达效，提升烟草、煤炭、冶金、化工等传统产业发展动能。坚持以供给侧结构性改革为主线，深入推进“三去一降一补”取得新进展。积极推进钢铁化解过剩产能工作，巩固煤炭去产能成果，稳妥推进煤矿复产复建工作，力争生产原煤3000万吨。进一步落实促进房地产业发展的政策措施，优化住房供给结构，加大棚户区改造货币化安置力度，化解商品房库存300万平方米。完善政银企常态化对接等5项机制，促进金融机构降低企业融资成本、服务实体经济，维护金融市场稳定。实行收费目录清单管理制度，规范行政审批中介服务收费，降低制度性交易成本；推动大型工商业用户和园区中小企业打包参与电力市场化交易，将电力优势转化为产业优势；推行工业用地出让弹性年期制和先租后让、租让结合供应方式，建立铁路运输量价捆绑、阶梯运价合作机制，进一步落实税收优惠和降低、缓交社会保险费政策，打好降低企业成本“组合拳”。抓实补短板重点项目建设，努力破解制约经济社会发展的瓶颈。

　　培育经济发展新动能。围绕重点发展的6大产业，继续实施“十、百、千”项目投资计划，力争产业投资、民间投资分别占固定资产投资的40%、45%，制造业投资占产业投资的35%。着力发展高原特色农业及生物资源加工产业。启动牡丹籽高端食用油、姜辣素精深加工等项目建设，推进魔芋深加工、紫杉醇生产等项目建设，确保马铃薯深加工、肉牛屠宰加工等项目建成投产，大力发展生物医药和特色保健食品产业，推动农产品加工转化率提高到56%，实现高原特色农业及生物资源加工产业增加值387亿元。着力发展有色金属深加工及液态金属产业。推进沾益白水铝产业园、富源中安铝加工园、摩托车轮毂制造、多晶硅及下游产品加工等项目建设，重点围绕液态金属产业引进一批重大项目落户，实现有色金属深加工及液态金属产业增加值55亿元以上。着力发展军民融合现代装备制造产业。启动军民融合产业示范园、现代装备制造技术孵化中心等项目建设，推动新能源汽车、轨道交通产业园等项目建设。支持一汽红塔汽车产品升级和市场开拓。加大电子信息产业招商引资力度。实现军民融合现代装备制造产业增加值20亿元以上。着力发展精细化工产业。推进煤制烯烃及煤制气等项目前期工作，启动米高集团二期硫酸钾项目建设，加快粗酚、二萘酚等项目建设，实现精细化工产业增加值117亿元。着力发展现代商贸物流产业。推进万达城市综合体等15个商贸物流项目建设。推广富源营上镇全省“七彩乡村购物”试点经验，建设一批乡村新型商业中心。培育和打造一批物流园区、大型专业市场、商贸综合体。实现现代商贸物流产业增加值314亿元。着力发展文化旅游和高原体育产业。抓好9个旅游融合发展示范区、31个旅游重大项目建设，做好1个国家5A级景区、4个4A 级景区、6个3A级景区创建工作，完善旅游公路、厕所、停车场等基础设施。推进罗平县全国全域旅游示范区创建。积极培育特色山水、温泉康体、低空观光等旅游新业态，加大旅游宣传推介力度，确保国内外旅游者接待人数和旅游业总收入分别增长18%、30%。加快国际高原体育城建设，完善配套基础设施，精心组织世界铁人三项赛、世界女子网球巡回赛等国际国内赛事。实现文化旅游和高原体育产业增加值105亿元。

　　全力打好“三大战役”。大力发展园区经济。实施园区提升“五大工程”，提升园区经济综合竞争力。组建园区投融资平台，加大园区基础设施建设力度，完成投资40亿元。发挥重点企业的龙头作用，打造一批专业化、特色化产业园区。引导资金、技术、项目、人才等创新要素向园区集聚，推动曲靖经开区高新技术产业园和省级高新区建设取得实质性进展。建立园区动态调整和定期考核机制，激发园区竞相发展的活力。大力发展县域经济。抢抓沪昆高铁开通机遇，以麒沾马为核心，找准城市功能定位和产业发展方向，主动融入滇中城市群一体化发展，大力发展高新技术、装备制造、高原体育等产业，做强城市经济。依托县域资源禀赋和基础条件，因地制宜发展重点产业，督促每个县（市、区）各抓实10个以上投资上亿元的重点产业项目。加大政策支持和督查考核力度，对进入全省县域经济跨越发展先进县、进位县行列的予以重奖，推动各县（市、区）争先进位。大力发展民营经济。落实民营经济发展政策，实行领导干部挂钩联系民营企业和定期研究民营经济工作制度，支持企业家专心创新创业。积极发展混合所有制经济，推动国有企业（资本）与民营企业（资本）双向融合，提高PPP项目落地率，扩大民营经济的比重。实施中小企业成长工程和新一轮微型企业培育工程，确保新增规模以上工业企业50户。

　　（二）打好精准脱贫攻坚战，切实抓好“三农”工作

　　深入推进脱贫攻坚。紧盯“师宗县摘帽，88个贫困村出列、9.13万贫困人口脱贫”的年度目标任务，强化督查考核和责任追究，以铁的纪律保障脱贫攻坚取得新成效。市级财政安排扶贫专项资金3亿元，加大各类扶贫资金、财政涉农资金整合力度，确保各类扶贫资金投入达200亿元以上。大力推广“农业扶贫产业园区+龙头企业+合作社+基地+贫困户”等模式，让贫困户分享农业产业链和价值链增值收益。主攻易地扶贫搬迁、农村危房改造两大难点，抓好贫困地区基础设施建设，完成易地扶贫搬迁1.3万户4.9万人，其中建档立卡贫困户7787户2.6万人；实施农村危房改造及抗震安居工程3万户，其中建档立卡贫困户1.8万户。加大贫困地区农村青壮年劳动力技能培训力度，推动2.8万贫困人口转移就业。全面落实各项配套措施，确保生态补偿、教育支持、健康扶贫、社保兜底等脱贫措施真正落实到户到人。全力争取沪滇扶贫协作支持，开展“百企帮百村”精准扶贫行动，加强与中国农工民主党中央、中国工程院、中国航天科工集团等中央定点帮扶单位的精准对接，汇聚更多资源和力量向贫困决战。

　　积极推进农业现代化。把增加绿色优质农产品供给放在突出位置，狠抓农产品标准化生产、品牌创建、质量安全监管。实施2个国家级新增千亿斤和6个省级新增百亿斤粮食产能县工程，稳定粮食播种面积，加强市级粮食储备库建设，确保粮食安全。着力优化产业产品结构，推进现代烟草农业提质增效，提高油菜、水果、花卉等8大基地建设水平，发展特色经济作物632.5万亩，新建“百千万”畜禽养殖示范场40个。推进联想佳沃蓝莓、广东温氏等5个农业园区建设，推动农村一二三产业融合发展。发展适度规模经营，大力培育新型农业经营主体，新增土地承包经营权流转30万亩，新增固定资产投资500万元以上的农业龙头企业15个、市级合作示范社18个。

　　着力夯实农业发展基础。继续推广陆良农田水利改革试点经验，建设高效节水灌溉农田20万亩，改造中低产田地20.6万亩。抓好第三次全国农业普查工作，推进陆良县国家农村产业融合发展试点和全省农业统计调查改革试点。深化农村产权制度改革，统筹推进农村土地征收、集体经营性建设用地入市、宅基地制度改革试点，完善集体林权制度，赋予农民更加充分的财产权利。深化供销社、国有林场等改革。扩大农业保险覆盖范围。

　　（三）加快城乡基础设施建设，推进城乡统筹发展

　　强化“五网”基础设施建设。建成沾会、东过境、宣曲、江召新建段、大瓦仓至昌隆铺高速公路，在全省率先实现“县县通高速”目标。推进沾益至寻甸、召夸至泸西、富源至兴义高速公路建设，开工建设三宝至清水、威宁至宣威、罗平至八大河高速公路，推进宣威至富源、曲靖至泸西、阿岗至罗平等高速公路前期工作。加快国道326线杉木箐至宣威段建设，完成国道213线会泽江底至钢厂段建设。新建、改建农村公路2000公里，实现建制村全部通硬化路目标。配合做好渝昆高铁建设，完成师宗至文山铁路可研报告编制工作。力争宣威支线机场和沾益、会泽、罗平通用机场开工建设，推进沾益通用航空基地建设。扎实抓好移民安置工作，加快阿岗水库建设，确保车马碧水库一季度开工，做好黑滩河水库项目前期工作，推进会泽白马箐、麒麟龙潭河等41件新建、续建骨干水源工程。新建城市天然气管网57公里，力争实现中心城区居民用户燃气全覆盖。完成新一轮农村电网改造升级项目1432个，新建、续建35千伏以上输变电工程14个。推动光纤宽带和4G通信网络向自然村延伸，力争城镇光纤接入能力达50M以上、行政村光纤接入能力达12M以上。积极争取纳入国家新型智慧城市建设试点，组建“智慧曲靖”运营平台公司，启动建设曲靖市信息中心，确保在智慧煤监、智慧旅游、智慧养老、智慧医疗、智慧公安、智慧交通等领域取得突破性进展。

　　坚持高起点规划。树立现代规划理念，更加注重保护自然生态与山水特色，更加注重传承历史文脉与历史记忆，更加注重提升城市品质与文化品位。开展麒沾马“多规合一”编制工作，推进中心城区公共服务设施、绿地系统、综合交通、地下空间综合开发利用等专项规划编制，力争控制性详细规划全覆盖。完善县域城乡规划体系，做精县域中心城镇，做特做靓小城镇。加强传统村落保护，优化提升民居方案，科学引导民居建设。制定城市设计导则，完成沾益区城市设计试点工作，开展优秀建筑、规划和城市设计方案评选，建设更加宜居、更有活力、更具特色的现代化城镇。

　　加快新型城镇化进程。深入推进国家新型城镇化综合试点，把促进有能力在城镇稳定就业和生活的常住人口有序实现市民化作为首要任务，健全农业转移人口市民化成本分担机制，加大土地、财政等领域配套改革力度，推动常住人口基本公共服务全覆盖，确保户籍人口城镇化率提高1个百分点。扎实开展师宗、马龙深化县城基础设施投融资体制改革试点，实现马龙撤县设区。深入推进麒麟越州镇、沾益白水镇“社区、产业园区”两区同建试点，打造一批特色小镇。

　　增强城镇承载能力。推进麒马大道、经沾路同城化道路建设工程，改造7条内部干道，打通7条断头路，建成5个公交场站，构建城市快速交通体系。启动城市轨道交通建设，带动轨道交通配套产业发展。完成7个排水防涝工程，加快城市新区地下综合管廊建设，同步引进建设运营中心。推进中心城区海绵城市示范区建设。改造提升寥廓公园，建成沾益西河公园，新建沾益玉林公园、马龙龙湖公园，做好第十一届中国（郑州）国际园林博览会曲靖园布展工作。推进北城新区历史文化旅游区建设。重点推动宣威南片区、陆良西片区、师宗南片区、罗平南北片区、富源东西两线、会泽蔓海公园片区建设，提升县城承载能力。

　　提升城乡管理水平。理顺城市管理综合行政执法体制，推进行政执法工作重心下移，明确管理范围、制定权责清单、落实责任主体，规范执法制度、提高执法素养、改进执法方式、完善监督机制，探索社区、居民参与管理机制，提升城市管理能力和服务水平。加强城市管理数字化平台建设和功能整合，建设综合性城市管理数据库。巩固国家园林城市创建成果，确保沾益、马龙达到国家园林城市标准，师宗创建为国家园林县城。深入推进城乡人居环境提升五年行动计划，扎实开展城市“四治三改一拆一增”和农村“七改三清”行动，坚决整治违章建筑、占道经营、乱停乱放等突出问题，实施农村环境整县、整乡综合整治工程，提升城乡“颜值”和品质。

　　（四）全面发展社会事业，扩大人民群众获得感

　　坚持教育优先发展。扎实推进区域教育中心建设，大力发展学前教育，均衡发展义务教育，优质发展高中教育，提升发展职业教育，积极发展民办教育，支持发展高等教育。加大标准化幼儿园建设力度，新增幼儿园学位2.35万个。改善200所义务教育薄弱学校基本办学条件，新增中心城区义务教育学位2.3万个，确保罗平、师宗通过县域义务教育基本均衡国家评估验收。探索教育集团办学模式，推动曲一中新校区建设，建成河北衡水中学曲靖分校。推进省级职业教育改革发展试验区建设，启动滇东职业技术学院、曲靖医药学院创建工作。加快教育信息化建设。推进教师“县管校聘”改革，探索建立教师岗位动态管理机制，落实乡村教师支持计划，持续实施“三名工程”和优秀骨干教师政府特殊津贴制度。

　　提高人民健康水平。扎实推进区域医疗卫生中心建设，普及健康生活、优化健康服务、完善健康保障、建设健康环境、发展健康产业，提高全民健康水平。新建2所县级综合医院、4个乡镇卫生院，提高基层医疗卫生服务能力。加快市中医院新院建设，确保市妇幼保健院新院建成投入使用。充分发挥市级三甲医院学科诊疗优势，推进7个特色临床专科集群建设。建立招聘高层次和紧缺人才“绿色通道”，加强医技人才队伍建设。推动中医药事业发展，确保创建为全国基层中医药工作先进市。做好公立医院改革试点工作，确保在完善医疗服务体系、改革医保支付制度、创新医院管理体制等方面取得突破性进展。坚持计划生育基本国策，认真贯彻落实“全面两孩”政策。抓好国家卫生城市复审命名工作，推进卫生县城、卫生乡镇和卫生村创建活动，确保沾益、马龙达到国家卫生城市标准。

　　健全社会保障体系。做好高校毕业生、化解过剩产能分流人员等重点群体就业创业工作，扶持创业1.5万人，新增城镇就业3.6万人，农村劳动力转移输出就业15万人。落实养老保险制度改革政策，巩固全民参保登记工作成果，推动社保由制度全覆盖向人员全覆盖转变。完善社会救助制度，提高社会救助整体水平。推进师宗“两线合一”工作。扎实推进全国养老服务业综合改革试点工作，大力发展健康养老产业，加快市级养老综合服务中心和30个社区（村）居家养老服务中心建设，扶持10个以上民办或公建民营养老机构，改扩建10所农村敬老院。扎实推进殡葬改革，完成曲靖殡仪馆迁建，新建富源县殡仪馆，建成81个乡镇级农村公益性公墓。

　　弘扬社会主义核心价值观，完善政府向社会力量购买公共文化服务机制，推动城市15分钟、乡村半小时文化体育服务圈建设，加强历史文物古迹和民族民间文化保护，提升文艺作品创作能力，开展多层次群众文化体育活动，确保国家公共文化服务体系示范区创建通过验收。全面完成会泽地震灾区灾后恢复重建工作。落实党的民族宗教政策，深化民族团结进步示范区创建活动，发挥宗教界爱国人士和信教群众在促进经济社会发展中的积极作用。加强国防后备力量建设，做好国防动员、民兵预备役和人民防空工作。统筹做好侨务、地震、档案、保密、地方志、社会科学等工作，推进红十字、慈善等事业发展。

　　（五）深化改革创新，激发经济发展活力

　　深化重点领域改革。树立“特区思维”，推动以服务企业为核心的体制机制创新，营造良好的营商环境。继续加大简政放权力度，确保政府公布的行政审批事项全部入驻政务服务中心办理。加大公共资源电子化交易平台建设，确保实现“网下无交易、网上全公开”目标。深化商事制度改革，探索推进“多证合一”。全面推行“双随机、一公开”监管机制，完善社会信用信息管理体系，扩大信用评价结果运用领域。落实税制改革政策，巩固“营改增”成果。加强预算管理，推进财政事权与支出责任划分改革。探索财政涉企资金基金化改革，组建市级重点产业发展基金。积极推进国企改革，整合政府资产和各类资源，壮大政府投融资平台实力。支持曲靖商业银行引进战略投资者增资扩股，确保2家县级农村信用联社改制组建为农村商业银行，新增“新三板”挂牌企业2户以上，创建信用乡（镇、街道）12个。完成承担行政职能的事业单位改革试点。完善不动产统一登记体系。深入推进公务用车制度改革。推行机动车驾驶证自学直考改革。

　　构建科技创新体系。以企业技术开发为主体、院士（专家）工作站为支撑、高等院校及众创空间为补充，构建具有曲靖特色的开放式科技创新体系。组建科技创新基金，新建院士（专家）工作站5个，新增国家高新技术企业20户，认定省级科技型中小企业80户、市级以上企业技术中心12个。建设曲靖产业技术研究院，支持高等院校建设一流大学、一流学科。支持麒麟区申报国家级高新技术开发区，加快清华启迪科技园建设，建成国家级南高丛蓝莓工程技术研究中心。支持创新创业平台建设，规划建设一批人才公寓和创新社区，探索全天候或候鸟式的人才引进方式。启动全国质量强市示范城市创建，抓好重点产业产品标准制定和修订工作。

　　提升开放合作水平。广建“朋友圈”，深化与深圳、金华等友城在产业转移、科技教育、文化体育等方面的合作，争取在缔结国际友好城市上取得突破。承办好世界蓝莓大会，建设曲靖南亚东南亚经贸文化交流中心。积极承接发达地区产业转移，瞄准长三角、珠三角、环渤海和欧美发达地区，大力开展行业招商、专业招商、精准招商、以商招商，高度重视全产业链招商，确保实际到位市外国内资金突破960亿元，增长8%，确保引进1户以上境外世界500强企业。全力推进曲靖综合保税区申报工作，推动保税仓库建设，力争外贸进出口总额突破7亿美元，增长12%。

　　（六）强化生态文明建设，推动绿色可持续发展

　　落实最严格的环保制度。牢固树立“绿水青山就是金山银山”的理念，积极构建政府、企业、公众共治的环境治理体系。紧扣中央环保督察组反馈问题，全面清理排查污染源和环境安全隐患，逐项建立责任清单，坚决整改存在的突出环境问题。确保危险废物集中处置项目建成投入使用。建立重大项目环境专家咨询制度，严格执行规划环评，落实生态环境保护责任清单，实行环保重大决策终身责任追究及责任倒查制度，严守“三线一单”硬约束。积极推进环保机构监测监察执法垂直管理改革试点，加强环境监管能力建设。强化环境监管网格化管理，推行环境污染第三方治理，提高治污水平。

　　强化生态环境治理。开展大气污染防治专项整治行动，启动市环境空气质量预测预报系统建设，建立污染天气会商制度和重污染天气应急机制，开展中心城区空气质量源解析工作，加大城市扬尘、工业集中区、机动车排气、噪声等污染防治力度。认真落实“河长制”，加强饮用水源地保护，做好南盘江、北盘江、牛栏江等重点流域水污染防治，确保出境断面水质基本达标。划定土壤环境保护优先区域及重点治理区，实施土壤环境质量评估和污染源排查，加大重金属污染治理力度。深入推进农业面源污染综合治理、病死畜禽无害化处理工作。加快工业园区集中式污水处理设施和85个小城镇“一水两污”设施建设，确保中心城区南片区污水处理厂投入使用。

　　推进“森林曲靖”建设。全面完成三年城乡绿化攻坚行动，实施生态修复、森林资源保护、城镇绿化等“6大工程”，推进国家森林城市创建。大力实施公益林保护、木本油料建设和退耕还林还草、陡坡地治理工程，完成营造林60万亩，治理水土流失面积500平方公里。提升森林资源管护水平，强化湿地和自然保护区监管，加大森林防火力度，提高林业有害生物防治水平，严厉打击破坏森林资源违法行为，确保森林覆盖率达47%。

　　节约集约利用资源。推进国家循环经济示范市创建工作，推广循环型生产方式、流通方式和绿色消费模式。实施20项市级节能示范项目，安装太阳能热水器4000台、节能改灶4000户。坚守耕地红线，全面划定永久基本农田，持续推进低丘缓坡土地综合利用试点，加大工矿废弃地复垦力度，大力盘活闲置土地，节约集约利用土地资源。全面落实最严格水资源管理制度，推进4个省级最严格水资源管理制度示范县和2个省级节水型社会示范县创建工作。

　　（七）从严落实责任，全力维护社会和谐稳定

　　强化安全生产监管。严格落实“党政同责、一岗双责、失职追责”和“管行业必须管安全、管业务必须管安全、管生产必须管安全”的安全生产监管责任。深入开展重点行业领域安全生产专项整治，强化企业安全生产主体责任，着力堵塞监督管理漏洞，着力解决不遵守法律法规的问题，坚决杜绝重特大事故，有效遏制较大事故，最大限度减少一般事故，确保安全生产形势持续稳定好转。全面贯彻落实“四个最严”要求，推进国家食品安全城市和农产品质量安全示范县创建工作，加强食品药品检验检测和信息化监管能力建设，建成食品生产加工小作坊园区10个、标准化食用农产品交易市场20个、食品摊贩集中生产经营区20个，确保70%以上的乡镇建成市级食品药品安全乡镇。

　　提高社会治理能力。加强信访基础业务规范化、信访信息化建设，畅通群众诉求表达、利益协调和权益保障渠道。严格落实属地管理责任和部门事权管理责任，积极推行诉访分离，深入开展领导干部“大接访、大下访”活动，切实解决好人民群众的合理合法诉求。深入推进“平安曲靖”建设，加强综治基层基础工作，打好禁毒防艾人民战争，依法严厉打击危害人民群众生命财产安全、危害社会治安、危害公共安全的违法犯罪活动，不断提高人民群众的安全感。推进“七五”普法，加强社区矫正和安置帮教工作，抓好人民调解工作，提高法律服务水平。完善舆情工作机制，主动回应媒体关切，做好突发事件应急处置工作。

　　（八）坚持全面从严治政，努力建设人民满意政府

　　增强“四个”意识，牢记发展使命。进一步增强“四个意识”特别是核心意识、看齐意识，更加紧密地团结在以习近平同志为核心的党中央周围，更加坚定地维护以习近平同志为核心的党中央权威，更加自觉地在思想上政治上行动上同以习近平同志为核心的党中央保持高度一致，更加扎实地把党中央国务院、省委省政府和市委的各项决策部署落到实处，担好深化改革之责，履好推动发展之责，负好服务民众之责。坚持政府党组中心组学习制度，补齐知识缺失、能力不足、本领恐慌的短板，培养专业思维、专业素养、专业方法，提高领导经济发展的能力和水平。继续开展“狠抓落实年”活动，建立健全重大决策部署分解、督办、反馈工作机制，推动重点项目、重大事项、重要工作快谋划、快推进、快落实。完善绩效管理和综合考核评价体系，充分发挥督查考核的“风向标”“指挥棒”作用，切实解决“为官不为”的问题，督促各级领导干部多谋打基础、添后劲、利长远的实举，多办顺民意、惠民生、得民心的实事，多出经得起实践、人民、历史检验的实绩。

　　严格依法行政，建设法治政府。严格执行政府系统领导干部宪法宣誓制度，增强职业使命感和责任感。严格落实立法法规定，提高政府立法质量。加强规范性文件监督管理。推进曲靖智库建设，落实公众参与、专家论证、风险评估、合法性审查、集体讨论决定的重大行政决策法定程序，推进重大决策科学化、民主化。全面落实行政执法责任制，完善行政执法程序，创新行政执法方式，提高政府工作人员法治思维和依法行政能力。全面推进政务公开，自觉接受人大的法律监督、工作监督和政协的民主监督，虚心接受社会监督和舆论监督，认真办理人大代表议案、建议和政协提案。

　　守住纪律底线，加强廉政建设。把全面从严治党要求落实到从严治政中，严格遵守政治纪律和政治规矩，认真贯彻执行《关于新形势下党内政治生活的若干准则》和《中国共产党党内监督条例》，切实履行“一岗双责”，落实全面从严治党主体责任和监督责任，扎实推进政府系统党风廉政建设和反腐败斗争。巩固“两学一做”学习教育成果，认真落实中央“八项规定”精神，坚决反对“四风”，严肃查处违纪违法行为。加强对政府购买公共服务、政府基金项目、政府债务的管理，紧盯行政审批、工程建设、惠民资金等重点领域和关键环节，强化行政监察和审计监督。牢固树立过紧日子的思想，压缩“三公经费”支出，把有限的资金用在刀刃上。

　　各位代表，奋斗成就梦想，实干铸就辉煌。让我们紧密团结在以习近平同志为核心的党中央周围，在省委、省政府和市委的坚强领导下，牢记使命、勇于担当，扎实苦干、奋发有为，奋力开创曲靖跨越式发展新局面，在全面建成小康社会进程中走在全省前列，以优异成绩迎接党的十九大胜利召开！
